# Editorial: Cardiovascular imaging in the integrated assessment of metabolic health

**DOI:** 10.3389/fcvm.2023.1276182

**Published:** 2023-08-30

**Authors:** Helena B. Thomaides-Brears, Rajarshi Banerjee

**Affiliations:** Perspectum, Translational Science, Oxford, United Kingdom

**Keywords:** diabetes, quantitative imaging, long COVID, integrated assessment, value-based healthcare, atherosclerotic vascular disease, multiorgan acquisition

**Editorial on the Research Topic** Cardiovascular imaging in the integrated assessment of metabolic health

Personalizing cardiovascular risk management is of utmost importance in multisystem conditions and may require multiple techniques to understand and treat the underlying pathophysiology, including quantitative imaging. This has been particularly relevant in the wake of COVID-19, which has highlighted a key need for further integrative medical approaches to diagnostics especially in individuals with pre-existing metabolic risk (1). The main aim of this Research Topic is to collate original research articles and reviews that use multi-modal imaging to provide insight into metabolic diseases that present with both organ-specific and extra-organ manifestations and require integrated care.

Murata et al. evaluate the level of permanent cardiovascular damage using multi-modality imaging in patients hospitalised for COVID-19 who displayed cardiovascular disease (CVD) symptoms as outpatients. In their local clinical cascade electrocardiograms (ECG), chest x-ray and echocardiography were applied to screen for atrial or ventricular involvement followed by cardiac magnetic resonance (CMR) or computed tomography (CT) to confirm findings. With this tiered strategy they ruled in the presence of cardiovascular disorders in 27% and found independent associations between a severe course of COVID-19 or in-hospital cardiac events with continued cardiovascular damage post-COVID.

There is further compelling evidence that convalescent COVID-19 patients have a heightened risk of CVD even one year after the initial infection. Borlotti et al. specifically review the value of CMR in understanding the long-lasting clinical consequences for patients hospitalised for acute COVID-19, in the community amongst individuals with a mild course of COVID-19, in athletes and as a rare adverse effect post anti-COVID vaccination. The most common abnormal finding on CMR speaks to the unique contribution of this technique: increased native T1 value in the myocardium, suggestive of underlying diffuse myocardial inflammation, even in the absence of elevated circulating biomarkers like hsTnI and NTproBNP. While evidence gaps remain, due to the suddenness of the pandemic and the ongoing virus evolution, the authors identify a place for CMR in long-term follow-up data collection. Contributing to the prismatic nature of long COVID syndrome were increased T1 relaxation times in organ parenchyma beyond the heart: in liver, kidney and pancreas identified in several studies exploring MRI phenotypes across multiple organs.

Integration of circulating biomarkers with imaging already has a place in care for established metabolic diseases. Naami et al. review the relationship between lipoprotein A [Lp(a)], a serum biomarker for familial hypercholesterolemia and genetic predisposition to atherosclerotic vascular disease (ASCVD), and coronary calcium calcification (CAC), a CT-imaged measure of calcium levels diagnostic of arterial plaque formation. The authors weigh out the potential benefits of a dual risk stratification strategy for ASCVD. Recent studies of larger cohorts indicate that in combination these tools are strong predictors of future ASCVD events. The authors argue that both Lp(a) and calcification independently contribute to ASCVD, but that evidence is required, in individuals without ASCVD risk factors and lower levels of LpA but high CAC, to specify the exact algorithm for use of both these tools in ASCVD clinical care. This ties well with recent European Atherosclerosis Society guideline recommendations for more intensive risk factor management with increasing Lp(a) concentration (2), which should be made possible once findings from current Lp(a)-lowering drug trials read out.

Wamil et al. review the CVD phenotypes associated with diabetes and explore evidence of diagnostic and clinical utility for different imaging modalities. They highlight that multiple left ventricular phenotypes prevail in diabetes, namely diastolic dysfunction, systolic dysfunction, myocardial changes, remodelling, ischemia and fibrosis, and require an array of imaging techniques: ECG, echocardiography, CT, MRI and myocardial perfusion scintigraphy. In asymptomatic patients with diabetes, the majority have some coronary artery disease (1 in 6 at time of diagnosis), at levels comparable to patients with previous myocardial infarctions*.* Echo is the most comprehensive and cost-effective option but only CMR (native T1 mapping and late gadolinium enhancement) can detect myocardial fibrosis. The authors propose a model in which AI-assisted ECG scoring systems and the CAC provide initial assessment of CVD risk, and stress echocardiography and perfusion scintigraphy or perfusion CMR could be added as first line tests to detect haemodynamically significant coronary artery disease or left main stem disease. In patients at higher risk of heart failure, ECG and echocardiography would be used as the first line investigations followed by CMR. They note that multi-organ MRI allows for integrated care across specialties. This is supported by the recent identification of diabetes subtypes with different comorbidity risks and trajectories (3).

The accepted standard for investigation of many pathologies is histological assessment after biopsy, in the endomyocardium, the liver or the pancreas. However, quantitative imaging provides a robust alternative with improved technical performance and objectivity, especially when complemented by other less-invasive wet lab tools. The multisystem nature of metabolic disease puts multiorgan imaging front and centre for integration of care to avoid putting patients at risk of the complications associated with biopsy- beyond long COVID and diabetes. This is underscored by the significant associations between imaging phenotypes of the heart, brain, and liver (4) and between an MRI biomarker of early liver disease activity and cardiovascular hospitalisation (5) found in the large-scale adult populations of the UK Biobank. The absence of an equivalent association with blood biomarkers in the liver, validates the need for imaging-based diagnosis, including provision of mechanistic insights [for example, clonal haematopoiesis as the root cause of the inflammatory response in the liver (6)]. The advent of quantitative accurate non-invasive imaging for the liver has accelerated research, revealing at least one modifiable risk factor for heart disease, just as it did for understanding of cardiac pathology two decades ago.
